# Evaluation of the Persian Version of Maslach Burnout Inventory-Human Services Survey among Iranian Nurses: Validity and Reliability

**DOI:** 10.22086/gmj.v0i0.995

**Published:** 2018-10-20

**Authors:** Saba Moalemi, Zahra Kavosi, Najimeh Beygi, Azizallah Deghan, Aliasghar Karimi, Mohammad Mahdi Parvizi

**Affiliations:** ^1^Student Research Committee of Shiraz University of Medical Sciences, Shiraz, Iran; ^2^Health Human Resources Research Center, School of Management & Information Sciences, Shiraz University of Medical Sciences, Shiraz, Iran; ^3^Noncommunicable Diseases (NCD) Research Center, Fasa University of Medical Sciences, Fasa, Iran; ^4^Student Research Committee of Fasa University of Medical Sciences, Fasa, Iran; ^5^Molecular Dermatology Research Center, Shiraz University of Medical Sciences, Shiraz, Iran

**Keywords:** Nurses, Job, Maslach, Burnout, Inventory, Human Services, Persian version

## Abstract

**Background:**

Nursing is a critical job in the health care system. However, nurses suffering from poor job conditions suffer from job dissatisfaction, eventually causing burnout. This is a very important concern for the health care system because the turnover of nurses leads to a waste of money and time of this system. Therefore, nurse managers need to find a way to measure and reduce the burnout. The Maslach Burnout Inventory-Human Services Survey (MBI-HSS) is a famous inventory to measure the job burnout in human services. This study aimed to measure the validity and reliability of the Persian version of MBI-HSS.

**Materials and Methods:**

This study was conducted in two hospitals of Fasa University of Medical Sciences, Fars Province, Iran, in July 2017. Nurses participated with their own discretion in this study and filled the MBI-HSS themselves. The questionnaire consisted of 22 items comprising three dimensions. Exploratory factor analysis and Cronbach’s alpha were performed in this study using Stata software, version 12.

**Results:**

Overall, 200 nurses were included in this study, with a mean age of 29.48 ± 6 years. The result of the exploratory factor analysis showed that the weight of each item in its own dimension was greater than 0.4 or another dimension. Also, the Cronbach’s alpha for 3 dimensions was greater than 0.7.

**Conclusions:**

Our study showed that the Persian version of MBI-HSS has sufficient validity and reliability, similar to that of the original version, for the measurement of burnout in Persian speakers of human services workers.

## Introduction

Nurses have an important role in the health system, and they help patients to recover soon and lead a normal life. Also, nurses can improve the patient’s quality of life and help them feel better, and therefore, nursing is crucial for society. However, this job is very stressful and difficult, and nurses tolerate tremendous mental and emotional pressure. Ultimately, this job leads to dissatisfaction among nurses and predisposes them to burnout [1, 2]. This is a global concern because the turnover of nurses results in increased costs and has an adverse effect on the health care system [3, 4]. The World Health Organization reported that burnout may spread around the world in the future [5].

In the past, studies showed that high workload and loss of social support at work is associated with burnout [6, 7].

The measurement of burnout is interesting for researchers and organizations so that many researchers attempted to investigate and present a way to evaluate it [5].

Herbert Freudenberger is a pioneer in the study of job burnout. He evaluated the psychological aspect of burnout based on symptoms of volunteer healers. He described burnout as disability and fatigue due to working more than an individual’s capacity. According to Freudenberger, burnout is a syndrome consisting of fatigue, forgetting personal needs, commitment to an exterior factor, working hard for a long time, personal pressures, the brute pressure in the team, and paying too much attention to the needs of the client [8]. This definition comprises only one dimension of burnout, and Freudenberger’s description emphasizes emotional exhaustion (EE) only [9].

However, further research showed the burnout has multi-dimensions, and depersonalization (DP) and reduced personal accomplishment (PA) play a role in burnout [10-12].

Maslach et al. presented a multi-dimensional questionnaire on burnout with psychological variables consisting of EE, DP, and reduced PA [13] and named it the Maslach Burnout Inventory.

Initially, this questionnaire was developed for human services workers (MBI-Human Services Survey [MBI-HSS]). Further, it was developed for the general population, other workers (MBI-general), and educators (MBI-ED) [13]. Many studies were performed using MBI, and this inventory was translated into some other languages [2, 14-17]. The validity and reliability of MBI-general and MBI-ED in the Persian language were measured. However, there is no study about the validity and reliability of MBI-HSS in the Persian language. In this study, we measured the validity and reliability of the Persian version of MBI-HSS in nurses who work in educational hospitals.

## Methods and Materials

This study was conducted in 2 hospitals of Fasa University of Medical Sciences, Fars Province, Iran, in July 2017. The age range of the participants was from 20 to 55 years, and each of them had worked for more than a year in the hospital as a nurse. They gave their consent to participate in this study and filled the questionnaire themselves.

MBI-HSS has 22 items that are categorized into 3 groups: EE (9 items), DP (5 items), and PA (8 items). The answers for each item range from 0 to 6 (0 = never, several times = 1, once a month = 2, several times a month = 3, once a week or less = 4, several times a week = 5, and everyday = 6).

Finally, the result of this questionnaire included three scores of EE, DP, and PA for each participant. If they received a high score in EE and DP and a low score in PA, they were considered to have a high level of burnout.

MBI-HSS was translated into the Persian language by a person who held a master’s degree in translation. Subsequently, the Persian and original versions were evaluated by 3 expert physicians.

### Statistical Analysis

Exploratory factor analysis is an analytical, multivariable method, which was used to investigate the dimensions of MBI-HSS.

Dimensions in the original version of the questionnaire were compared to the Persian version using exploratory factor analysis.

In addition, varimax rotation was used to identify the factors better. The minimal weight factor for each item was 0.4 [18].

In this study, the Spearman rank correlation coefficient was used to evaluate the construct validity, convergent validity (CV), and discriminant validity (DV).

CV denotes a relatively strong correlation between items in a dimension and the total score of the same dimension. DV denotes a weak correlation between items in a dimension with others. The correlation of an item with another dimension must be less than with its dimension, and if the correlation is less than 0.4, it implies that there is good CV [19, 20].

Cronbach’s alpha was performed to measure the reliability of the Persian version of MBI-HSS.

Cronbach’s alpha greater than 0.7 is acceptable, greater than 0.8 is good, and greater than 0.9 means excellent reliability [21].

All statistical analyses of this study were performed using Stata software, version 12 (StataCorp LLC, TX, USA).

## Results

A total of 200 nurses, including 147 (73.5%) females and 53 (26.5%) males, with the mean age of 29.48 ± 6 years participated in this study.

The mean experience work was 5.86 ± 5.04 years. More demographic data are presented in Table-1.

**Table 1:** Characteristics Data of Subject Study

**Age, mean ± SD**	29.4 ± 6
**Sex, n (%)**	
Male	51 (25.5)
Female	146 (73)
**Marital status, n(%)**	
Single	68 (34)
Married	127 (63.5)
**Number of children, mean ± SD**	0.5 ± 0.8
**Years of work experience, mean ± **SD	6.1 ±6.4
**Education level, n (%)**	
Bachelor’s degree	192 (96)
Master’s degree	6 (3)
**Employment Status, n(%)**	
Official	50 (25)
Contractual	60 (30)
Obligation	62 (31)
**Residency, n(%)**	
In the city of work	148 (74)
Another city	20 (10)

###  Exploratory Factor Analysis

The result of varimax rotation for factor analysis shown in Table-2, and it identified three dimensions by weight of factor.

**Table 2:** Factor Analysis with Varimax Rotation of Items in Three Dimension

**Item**	**Rotated Component Matrixa**		
	**PA**	**EE**	**DP**
**1**	-0.012	0.784*	0.150
**2**	0.005	0.792*	0.111
**3**	0.010	0.767*	0.230
**4**	0.544*	0.233	- 0.226
**5**	-0.097	0.038	0.652*
**6**	-0.060	0.552*	0.438
**7**	0.675*	0.173	-0.178
**8**	0.006	0.743*	0.252
**9**	0.623*	-0.013	-0.046
**10**	-0.169	0.132	0.691*
**11**	0.016	0.344	0.560*
**12**	0.481*	-0.460	-0.048
**13**	-0.011	0.576*	0.418
**14**	-0.206	0.633*	0.206
**15**	-0.211	-0.024	0.664*
**16**	-0.069	0.707*	0.048
**17**	0.687*	-0.173	0.007
**18**	0.689*	-0.183	-0.186
**19**	0.633*	0.024	-0.062
**20**	-0.093	0.646*	0.307
**21**	0.552*	-0.113	-0.082
**22**	-0.070	0.265	0.454*

**a** Rotation converged in 6 iterations. * Factor weight greater than 0.4 is significate.

The items 4, 7, 9, 12, 17, 18, 19, and 21 were in PA, the items 1, 2, 3, 6, 8, 13, 14, 16, and 20 were in EE, and items 5, 10, 11, 15, and 22 were in DP.

### Construct Validity

Table-3 showed the Spearman correlation coefficient between the items and the triple-dimension questionnaire; the correlation coefficient of each item with its own dimension was higher than 0.4 and other dimensions. The table also shows the CV and DV of the questionnaire. The summary of the results is shown in Table-4.

**Table 3:** CV and DV by Spearman’s Correlation Coefficient between Three Dimension and Items of MBI-HSS (Persian Version)

**Dimension items**		**PA**	**EE**	**DP**
**EE**	1	-0.118	0.701*	0.332
**EE**	2	-0.088	0.690*	0.297
**EE**	3	-0.128	0.747*	0.367
**PA**	4	0.611*	-0.113	-0.214
**DP**	5	-0.289	0.446	0.704*
**EE**	6	-0.243	0.674*	0.435
**PA**	7	0.680*	-0.164	-0.297
**EE**	8	-0.130	0.719*	0.414
**PA**	9	0.661	-0.165	-0.198
**DP**	10	-0.360	0.464	0.762*
**DP**	11	-0.182	0.410	0.777*
**PA**	12	0.479*	-0.359	-0.318
**EE**	13	-0.229	0.703*	0.413
**EE**	14	-0.376	0.631*	0.515
**DP**	15	-0.425	0.457	0.647*
**EE**	16	-0.253	0.532*	0.448
**PA**	17	0.695*	-0.200	-0.272
**PA**	18	0.635*	-0.386	-0.347
**PA**	19	0.550*	-0.141	-0.153
**EE**	20	0.344	0.681*	0.403
**PA**	21	0.570*	0185	-0.269
**DP**	22	-0.251	0.427	0.550*

*CV: more than 0.4 0.4 and other dimension as mean strong correlation between items in a dimension

**Table 4:** CV and DV of Three Dimension of MBI-HSS

**Dimensions**	**Number of items**	**Range of CV**	**Range of DV**
**PA**	8	0.479- 0.695	-0.088 - 0.425
**EE**	9	0.532- 0.747	-0.113 - 0.646
**DP**	5	0.550- 0.777	0.153 - 0.515

### Reliability

The Cronbach’s alpha for all dimensions was greater than 0.7. Hence, it is a sign of internal consistency and reliability of MBI-HSS (Persian version).

Cronbach’s alpha of this questionnaire was 0.75, and the values for each dimension are shown in Table-5.

**Table 5:** Cronbach’s Alpha and Score of Three Dimension of MBI-HSS

**Dimensions**	**Number of items**	**Mean± SD**	**Cronbach’s alpha**
**MBI**	22	62.17± 16.37	0.75
**EE**	9	20.85± 11.82	0.85
**PA**	8	33.12± 8.69	0.76
**DP**	5	8.19± 6.33	0.71

## Discussion

Working conditions play an important role in job dissatisfaction for nurses [21]. These conditions include finances and occupational settings, and the adverse effect of on their lives [22].

In this study, the Persian version of MBI-HSS was evaluated and used to measure the burnout in 200 nurses of 2 hospitals.

Based on our results, the reliability of the Persian version MBI-HSS is similar to the original and other versions of MBI-HSS and approved that nurses are susceptible to burnout like previous studies [15-17]. Thus, the Persian version of MBI-HSS is reliable for the measurement of burnout in health care professionals.

The factorial analysis of this study identified the original version of MBI. Previous studies showed some problems with items 13 (feel frustrated) and 22 (feel recipients blame me), and they were not as per the expected factors [23].

Kim et al. [24] suggested removing items 2, 12, and 16. In addition, Richardsen et al. [25] and Schaufeli et al. [26] preferred to delete items 12 and 16. However, Lheureux [16] indicated to remove items 6, 12, 16, and 22 because these items were not suitable for the expected factors. Our result showed that the scores of 6 items included 0.55 for EE and 0.43 for DP. This suggested that this item had loaded on the unexpected factor. However, in the same condition, item 13 had more weight on the expected factor. Also, the factorial analysis of item 12 gave a score of 0.48 for PA and 0.46 for EE. These values were very similar, showing that this item loads on unexpected factor too. However, there is not a definitive conclusion concerning the issue of removing any item of MBI-HSS. This is challenging for researchers, and further investigation about this issue is needed.

## Conclusion

The Persian version of MBI-HSS has acceptable validity and reliability of the measurement of job burnout. Thus, we can use this questionnaire as a tool to measure the burnout of human services workers. The major limitation of this study was the low number of subjects; thus, to measure the burnout in Iranian nurses, this study must be performed in a larger population.

## Conflict of Interest

None declared.
